# Metallothionein expression in the central nervous system in response to chronic heavy metal exposure: possible neuroprotective mechanism

**DOI:** 10.1007/s10653-023-01722-5

**Published:** 2023-08-14

**Authors:** A. Navarro-Sempere, P. Martínez-Peinado, A. S. Rodrigues, P. V. Garcia, R. Camarinho, G. Grindlay, L. Gras, M. García, Y. Segovia

**Affiliations:** 1 Department of Biotechnology, Faculty of Science, University of Alicante, Apartado 99, 03080 Alicante, Spain; 2 Faculty of Sciences and Technology, University of the Azores, 9501-801 Ponta Delgada, Portugal; 3 IVAR, Research Institute for Volcanology and Risk Assessment, University of the Azores, 9501-801 Ponta Delgada, Portugal; 4 Centre for Ecology, Evolution and Environmental Changes and Azorean Biodiversity Group, CE3c, University of the Azores, 9501-801 Ponta Delgada, Portugal; 5 Department of Analytical Chemistry, Nutrition and Food Sciences, University of Alicante, PO Box 99, 03080 Alicante, Spain

**Keywords:** *Mus musculus*, MT2A, Neuroprotection, Volcanogenic pollution, Metal detection

## Abstract

It has been reported that volcanoes release several tonnes of mercury per year among other heavy metals through eruptions, fumaroles, or diffuse soil degassing. Since a high percentage of the world's population lives in the vicinity of an active volcano, the aim of this study is to evaluate the accumulation of these metals in the central nervous system and the presence of cellular mechanisms of heavy metal detoxification such as metallothioneins. To carry out this study, wild mice (Mus musculus) chronically exposed to an active volcanic environment were captured in Furnas village (Azores, Portugal) and compared with those trapped in a reference area (Rabo de Peixe, Azores, Portugal). On the one hand, the heavy metal load has been evaluated by analyzing brain and cerebellum using ICP-MS and a mercury analyzer and on the other hand, the presence of metallothionein 2A has been studied by immunofluorescence assays. Our results show a higher load of metals such as mercury, cadmium and lead in the central nervous system of exposed mice compared to non-exposed individuals and, in addition, a higher immunoreactivity for metallothionein 2A in different areas of the cerebrum and cerebellum indicating a possible neuroprotection process.

## Introduction

Geographical coexistence between humans and volcanoes is a reality with a growing trend. Currently, it is estimated that 14.3% of the world's population lives in the vicinity of an active volcano due to the benefits obtained from them: soil fertility, tourist attraction or the use of geothermal resources (Kelman & Mather, 2008; Linhares et al., 2015; Sigurdsson et al., 2015). However, inhabiting these environments involves risks to human populations that are not always related to explosive activity. Volcanoes are geological formations that, even when inactive or extinct, release pollutants into the atmosphere that are hazardous to human health, such as toxic gases or heavy metals (Bagnato et al., 2018; Ferreira et al., 2005), being mercury one of the main metals released (Edwards et al., 2021; Gustin et al., 2008; Selin, 2009). In nature, mercury is found in three different forms: mercury vapor or elemental mercury (gaseous elemental mercury, GEM or Hg^0^), inorganic mercury (I-Hg) and methylmercury (MeHg). GEM constitutes the > 90% if the atmospheric Hg and it is released by natural sources as volcanic systems and anthropogenic emissions (Liu et al., 2012a, 2012b). It remains in the atmosphere due to its stability and it can be oxidized to Hg^+2^ (Holmes et al., 2006), this I-Hg form is released to the atmosphere mainly by industry processes (Li & Tse, 2015) and it is deposited both in the land and water. There, I-Hg can be methylated to give rise to the organic form, MeHg, which its more concerning fate is its bioaccumulation in aquatic organisms or plants that constitute food for humans (Selin, 2009). The gaseous form is presumably the main route of entry to the organism of the inhabitants of volcanic environments (Camarinho et al., 2021).

Recent estimates indicate that volcanoes release between 45 and 700 tonnes of mercury per year, not only through eruptions, but also through fumaroles or diffuse outgassing zones (Pyle & Mather, 2003). Nriagu and Becker (2003) estimated that the worldwide flux of mercury from volcanic eruptions is 57 t/year, while the flux from degassing activities is 37.6 On the island of Sao Miguel (Azores archipelago, Portugal), volcanic activity is evident through hydrothermal manifestations such as cold CO_2_ and thermal springs, ground degassing and fumarole fields. The largest area of diffuse degassing on the island is located in the village of Furnas, a human settlement located in the caldera of the Furnas volcano, one of the three active volcanoes on the island together with Fogo and Sete Cidades. Living in these outgassing areas poses one of the greatest health hazards to the inhabitants due to the amounts of gases and aerosols that are continuously released; according to Viveiros et al., (2010) the Furnas volcano daily emits 968 tonnes of CO_2_. The emission of mercury in gaseous form from this volcano has also been studied. Bagnato et al. (2018) calculated that Furnas volcano emitted 9.6 × 10^–5^ t d^−1^ for an area of 0.04 km^2^. Given that the crater dimensions are 7 × 6 km, the amount of mercury vapor released each day is much higher. Therefore, it is possible that the inhabitants of this population may suffer health effects from chronic exposure to this metal, among other pollutants.

Gaseous elemental mercury has been shown to be able to cross the blood–brain barrier (BBB) and the placental barrier (Pamphlett et al., 2019; Solan & Lindow, 2014) and the exposure to this heavy metal has been linked to the development of neurodegenerative diseases (Bittencourt et al., 2022; Carocci et al., 2014; Corrêa et al., 2020; Farina et al., 2013; Luisetto et al., 2019). Its ease of crossing different cell barriers is due to its fat-soluble nature, which also allows Hg^0^ to cross the plasma membrane, accumulating inside the cells in the form of I-Hg (Cariccio et al., 2019). It has been reported in the literature that this form of mercury induces the production of reactive oxygen species (ROS) (Aragão et al., 2018; Monteiro et al., 2010; Sinha et al., 2013; Teixeira et al., 2018). Oxidative stress generated by ROS is especially harmful in the central nervous system (CNS) as it is a tissue that consumes high amounts of oxygen to carry out physiological functions, is largely composed of polyunsaturated fatty acids and the BBB impedes the passage of certain antioxidants such as vitamin E (Shukla et al., 2011). For these reasons, oxidative stress is considered a factor with a potential role in the pathogenesis of neurodegenerative disorders such as Alzheimer's, Parkinson's or amyotrophic lateral sclerosis (Block & Calderón-Garcidueñas, 2009; Niedzielska et al., 2016).

One mechanism that cells possess to reduce the amount of ROS generated are metallothioneins (MTs), low molecular weight proteins that are responsible for regulating the concentration of essential and non-essential metals, such as cadmium (II), mercury (I, II) and lead (II), as well as the homeostasis of copper (II) and zinc (II) (Martinez-Finley et al., 2012). In the presence of oxidative stress, MTs are rapidly translocated to the nucleus through nuclear pores, where they are oxidized and transported back to the cytosol (Nzengue et al., 2009). This system appears to be involved in protecting the cell against damage to genetic material and apoptosis.

In mammals, four isoforms named MT-I to MT-IV have been identified, of which MT-I and MT-II (known as MT-2A), are the most widely distributed; they are expressed in numerous cell types in different tissues (Babula et al., 2012). In the central nervous system, MT-I/II and MT-III isoforms not only have a different expression pattern, but also respond to different threats, with MT-I/II isoforms playing an important role in the overall CNS response to damage, however subtle (West et al., 2008). MT-I/II is located in both the cerebellum and spinal cord and is mainly expressed in astrocytes, especially those that have adopted a reactive form (Hidalgo et al., 2001).

Detoxification of metals as the main function of MTs has been extensively studied both in vivo (Kehrig et al., 2016; Montaser et al., 2010; Siscar et al., 2014; Yuvaraj et al., 2021) and in vitro (Hwang et al., 2013; Pirzadeh & Shahpiri, 2016; Qu & Waalkes, 2015; Shahpiri & Mohammadzadeh, 2018) and all isoforms of these metalloproteins are already considered as antioxidant elements.

Our previous studies have reported that mice chronically exposed to volcanic pollutants showed an accumulation of mercury in its inorganic form in the CNS, in different regions of the brain and spinal cord, including at the intracellular level in certain types of neurons of the dentate gyrus of the hippocampus (Navarro-Sempere et al., 2021b) and motor neurons of the ventral lumbar horn (Navarro-Sempere et al., 2022). In addition, changes in glial populations, such as astrocytes or microglia, have been described in the same hippocampal region. With regard to astrocytes, these animals showed an increase in the reactive form of these cells and astrocyte dysfunction related to a decrease in the enzyme glutamine synthetase (Navarro et al., 2021). On the other hand, the microglial population was also increased in these animals and reactive forms of microglia were found in the same region of the hippocampus. Likewise, we also observed an increase in the pro-inflammatory cytokine TNFα inside some neurons in the subgranular area of the dentate gyrus as well as in the polymorphic region (Navarro-Sempere et al., 2021a).

These previous results indicate that neuroinflammatory events are occurring in the CNS of these mice as consequence of neurotoxicity resulting from chronic exposure to volcanic contaminants. Although the presence of inorganic mercury in the CNS has been demonstrated in previous studies, it has not been quantified. In addition, volcanoes release other compounds that can accumulate in the CNS and cause other effects.

Finally, the main goal is to quantitatively analyze, for the first time, which elements accumulate in the CNS of mice chronically exposed to an active volcanic environment and to know whether these exposed organism display an increased expression of metallothioneins, especially isoform II, which could play an important role in anti-oxidation, anti-apoptosis and anti-inflammation processes in the CNS.

## Materials and methods

### Study areas and animal collection

The species *Mus musculus *(Linnaeus, 1758) was used as surrogate species in this study. Two groups of feral mice, *Mus musculus*, were captured alive in two different locations on the island of Sao Miguel. One group was captured in Furnas, a village with active volcanism (exposed group) and, another group was captured in Rabo de Peixe, a small village without active volcanism (reference group). Both locations are 24 km apart. The village of Furnas has a population of 1500 inhabitants and shows manifestations of active volcanism such as fumarole fields, CO_2_ hydrothermal springs and a marked soil degassing that contributes to a continuous input of volatile metals and other gases into the atmosphere. Rabo de Peixe, is a rural village of 5000 inhabitants with no evidence of active volcanism since the seventeenth century and no major sources of anthropogenic pollution. Furthermore, Rabo de Peixe is a coastal town and therefore has a high rate of air renewal.

Ten feral *Mus musculus* individuals (Furnas *N* = 5 and Rabo de Peixe *N* = 5) were captured alive by traps placed in different points of the villages and transferred alive to the laboratory in the shortest possible time, where they were anesthetized with isofluorane. Once a correct plane of anesthesia was reached, the animals were transcardially perfused with a saline phosphate buffer and a 4% PFA solution afterward. After perfusion, the brains were removed and fixed by immersion with 4% PFA overnight at 4 °C as described by Navarro-Sempere et al., 2021b. Of these brains, the right hemispheres were processed for light microscopy and the left hemispheres for metal quantification techniques. Parameters such as sex, weight and age were recorded for all individuals. Age was estimated from the dry weight of the lens as described by Quéré and Vincent (1989). Animals weighing less than 10 g were discarded from the study.

The experimental procedures were approved by the Ethics Committee of the University of the Azores (REF: 10/2020). All procedures were performed in accordance with the recommendations of the European Convention for the Protection of Vertebrate Animals used for Experimental and Other Scientific Purposes (ETS 123), the 2010/63 EU directive and the Portuguese decree law (DL 113/2013).

### Elemental analysis

A total of 12 elements (Al, Cd, Co, Cr, Cu, Fe, Hg, Mn Ni, Pb, V and Zn) were monitored in the brain samples. Apart from Hg, all the elements were determined by means inductively coupled plasma mass spectrometry (ICP-MS 8900, Agilent, USA) after a microwave-assisted digestion treatment (Ulltrawave, Millestone, Italy) following instructions reported in the Milestone Ultrawave application book. To this end, a certain sample amount (40–100 mg) was treated with 2.5 mL of concentrated nitric acid in a quartz tube using the digestion program recommended for biological samples. In all cases, the digestion was complete, and no solid residues were observed. Next, digested samples were diluted up to 5 mL and analyzed using acid matrix-matched standards. Experimental details for both ICP-MS determinations and sample digestion are gathered in Tables 1 and 2.

**Table 1:** ICP-MS operating conditions for the detection of dissolved ions and NPs

Plasma forward power (W)	1550
Argon flow rate (L min^−1^)	
Plasma	15
Auxiliary	0.9
Nebulizer (*Q*g)	1.00
Collision/reaction cell	He (4 mL min^−1^)
Sample introduction system	
Nebulizer	MicroMist® nebulizer
Spray chamber	Scott double pass
Sample uptake rate (*Q*l) (µL min^−1^)	300
Dwell time (ms)	100
Measuring time (s)	2
Replicates	3
Nuclides	^27^Al^+^, ^112^Cd^+^, ^59^Co^+^, ^52^Cr^+^, ^63^Cu^+^, ^56^Fe^+^, ^55^Mn^+^, ^60^Ni^+^, ^208^Pb^+^, ^51^V^+^ and ^66^Zn^+^

**Table 2:** Acid microwave-assisted digestion treatment program

Parameter	Setting
Digestion solution	5 mL HNO_3_
Sample mass/g	25–60 mg
Microwave power/W	1500
Ramp time/min	15
Temperature/ºC	170
Hold time/min	15

Because Hg is a highly volatile specie and could be lost during sample preparation, this element was directly determined in brain samples with the aid of a mercury analyzer device (DMA 80, Milestone, Italy) using a protocol described by the U.S. Environmental Protection Agency (EPA) in 2007. In this instrument, samples are deposited on quartz boats and heated under a control temperature program in the presence of oxygen. Next, pyrolysis products stream passes through a catalyzer and an Au-based trap where Hg is specifically retained in the form of amalgam. Finally, the trap is rapidly heated for Hg release and detection by means atomic absorption spectrometry. In this work, the standard temperature program recommended by the instrument manufacturer was employed without any further modification (Table 3). It is the oxidized form of Hg (Hg^2+^) that is detected with this methodology.

**Table 3:** Mercury analyzer temperature program

Parameter	Setting
Drying temp/time	60 s–200 °C
Decomposition Ramp	90 s–650 °C
Decomposition hold	90 s at 650 °C
Purge time	60 s
Amalgamation time	12 s
Recording time	30 s

Because there is no brain certified reference material (CRM) for trace metal analysis, a fish protein CRM (DORM-3, National Research Council, Ottawa, Ontario, Canada) was employed as a proxy to validate methodology traceability and trueness. This sample was analyzed using the above-mentioned procedures both before and after analyzing brain samples and no statistical differences were found regarding the certified values (*p* < 0.05, 3 replicates).

### Histological processing and immunofluorescence assay

From the right hemispheres of the tested samples, sagittal sections with 4 µm thickness were obtained using a microtome (Microm HM 340E). To perform the immunofluorescence assay, these sections were subjected to heat-mediated antigenic retrieval and blocked with 10% BSA for 90 min at room temperature. They were then immunolabeled with the primary anti-MT-2A antibody (DF6755, Affinity Biotech) at a 1:100 dilution overnight at 4 °C. The next day, the sections were washed and incubated with the secondary antibody (SAB4600310, Sigma Aldrich Co.) at a 1:500 dilution for 3 h at room temperature and shaking. They were then washed several times and mounted with Vectashield medium (Vector Laboratories, Burlingame CA) containing DAPI to stain the nuclei. For imaging, a Zeiss confocal microscope was used at 20× magnification. Microphotographs were taken every 0.5 µm in the Z-plane maintaining constant pinole, contrast and brightness, and an orthogonal projection of each slice was obtained using Zen Blue software. Three sagittal slices of each individual separated by 150 µm were studied.

### Statistical analysis

Differences in metal concentrations between samples from Furnas and Rabo de Peixe were analyzed. For this purpose, one-way permutational ANOVA (PERMANOVA) with Euclidean distance (McArdle & Anderson, 2001) was performed for each element (Cd, Hg and Pb) separately. In addition, p-value was calculated using Monte Carlo method (Anderson, 2014). PERMANOVA was conducted in PRIMERv6-PERMANOVA+ software package (Anderson et al., 2008).

Data regarding the age and weight of specimen captured in both study areas was compared using *t*-Students test, and a p-value less than 0.05 was considered as statistically significant. The software Graph Pad Prism (Graph Pad Software Inc., La Jolla, CA, USA) was used to conduct this statistical analysis.

## Results

All samples used in this study correspond to male individuals. No significant differences were found in the age of the animals caught in both study areas (Furnas: 234 ± 19 days and Rabo de Peixe: 292 ± 36 days; *p* = 0.191, Students' *t*-test) nor in body weight (Furnas: 15.18 ± 1.03 g and Rabo de Peixe: 13.4 ± 2.07 g; *p* = 0.124, Students' *t*-test).

### Elemental analysis

**Fig. 1:**
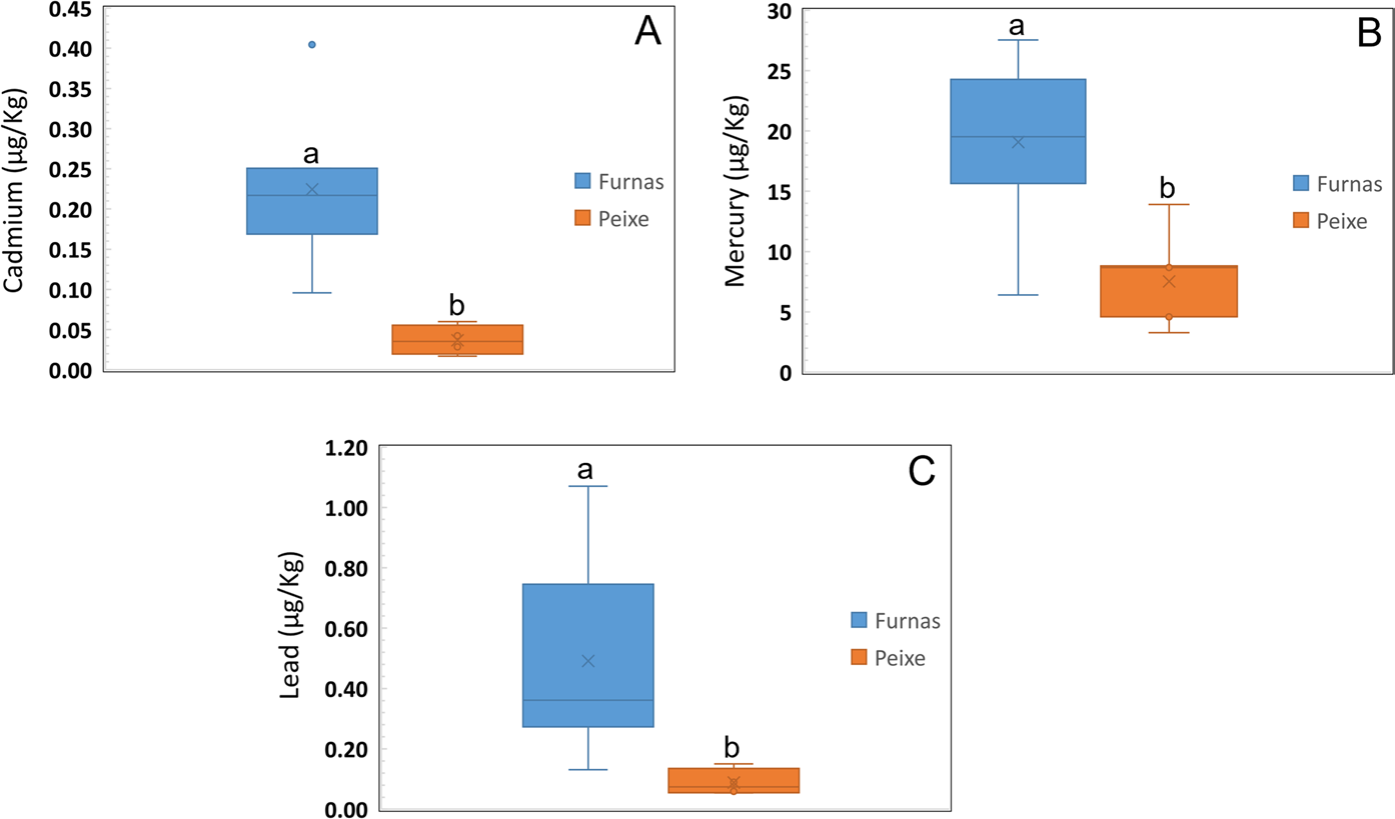
Metal concentration in mice brain from Furnas and Rabo de Peixe. Cd: Pseudo-F1,10 = 20.928, *p* value (MC) < 0.01 **a** Hg: Pseudo-F1,10 = 22.242, *p* value (MC) < 0.001 **b** Pb: Pseudo-F1,10 = 7.725, *p* value (MC) < 0.05 **c**. Line within the box, median; thin vertical lines, minimum and maximum values. Different letters over the bars indicate significant differences between sites

A total of 12 elements (Al, Cd, Co, Cr, Cu, Fe, Hg, Mn Ni, Pb, V and Zn) were determined in mice brain samples from both Furnas and Rabo de Peixe (Table 4). It was observed that Furnas mice samples show higher levels of Cd, Hg and Pb than those from Rabo de Peixe (Fig. 1a–c). The average concentrations for these elements in Furnas mice samples were, respectively, 0.22 ± 0.09, 19 ± 6 and 0.5 ± 0.3 g kg^−1^. For Rabo de Peixe, however, metal levels found were 0.04 ± 0.02, 8 ± 4 and 0.09 ± 0.04. Irrespective of mice location, the highest concentration found was for Hg, followed by Pb and Cd. Mercury concentrations in Furnas mice were especially high, ranging from 6.6 to 28 µg kg^−1^. For Rabo de Peixe mice, metal levels are mostly below 10 µg kg^−1^. Lead concentration ranges for Furnas and Rabo de Peixe were, respectively 0.13–1.1 and 0.05–0.15 µg kg^−1^. Cadmium concentration levels for Furnas samples ranged from 0.10 to 0.40 µg kg^−1^ whereas for Rabo de Peixe varied between 0.02–0.06 µg kg^−1^. Finally, no significant differences on metal concentration were observed for the remaining elements (Al, Co, Cr, Cu, Fe, Mn Ni, V and Zn) between mice samples from both locations.

**Table 4:** Results of the elemental analysis of mice brain samples and limits of detection. Concentration levels expressed in µg kg^−1^

Element	Furnas			Rabo de Peixe			
	Min	median	max	min	median	max	LoD (µg kg^−1^)
Al	2.4	3.4 ± 0.9	5.6	2.4	4 ± 3	7.0	0.04
Cd	0.10	0.22 ± 0.09	0.40	0.02	0.04 ± 0.02	0.06	0.004
Co	0.19	0.43 ± 0.17	0.6	0.12	0.6 ± 0.4	0.9	0.003
Cr	0.35	1.1 ± 0.5	2	0.3	1.4 ± 1.1	2.6	0.05
Cu	2.7	3.7 ± 0.7	4.8	2.6	4.5 ± 1.2	5.2	0.06
Fe	16	21 ± 4	29	20	24 ± 3	26	0.1
Hg	6	19 ± 6	28	3	8 ± 4	14	0.5
Mn	0.34	0.60 ± 0.19	0.90	0.47	0.60 ± 0.13	0.74	0.008
Ni	0.5	1.1 ± 0.7	2	0.3	0.7 ± 0.3	1.1	0.02
Pb	0.13	0.5 ± 0.3	1.1	0.05	0.09 ± 0.04	0.15	0.001
V	0.06	0.009 ± 0.004	0.013	0.07	0.013 ± 0.005	0.017	0.008
Zn	23	26 ± 3	31	21	24 ± 3	27	0.03

### Detection of metallothioneins in the CNS

**Fig. 2:**
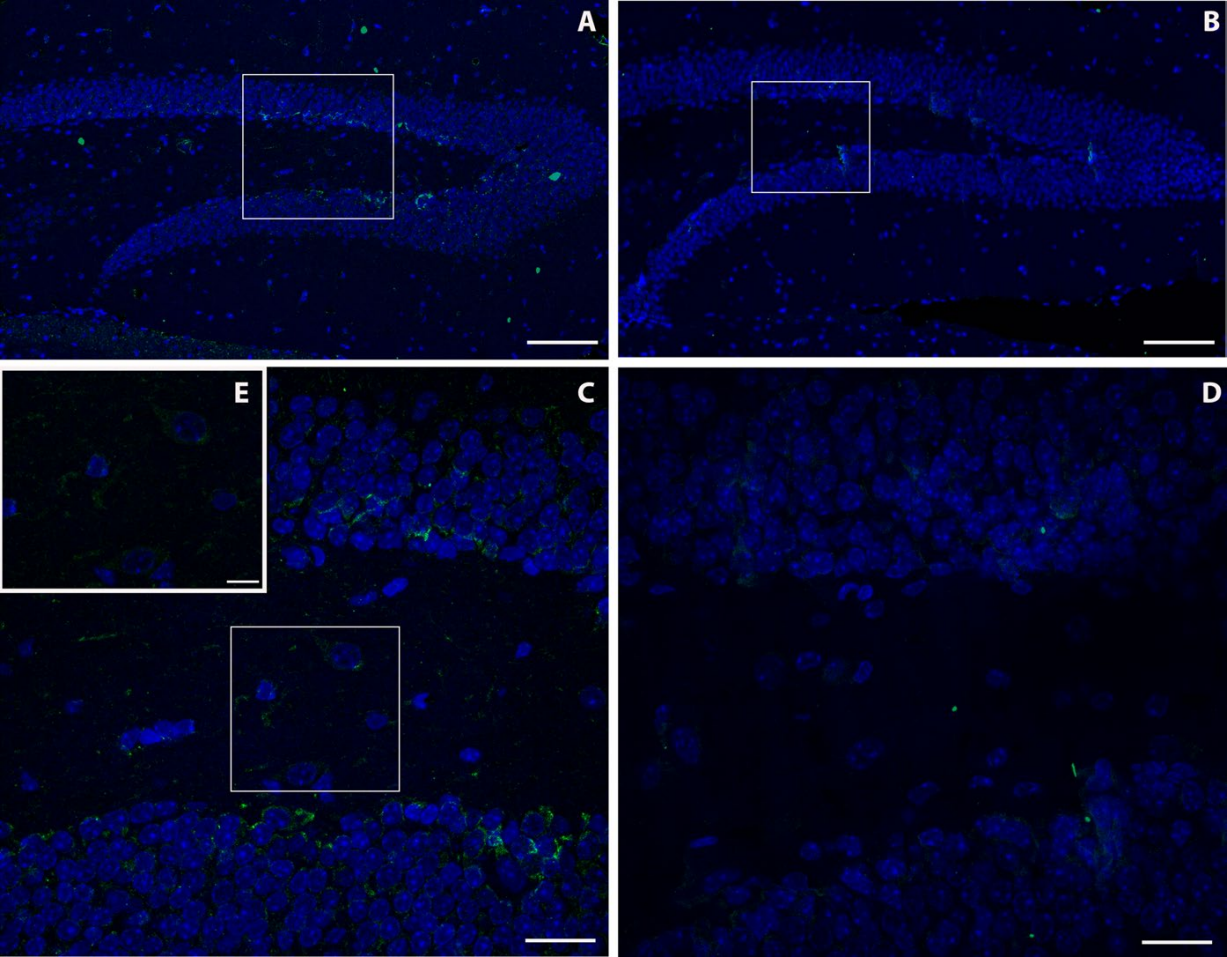
Immunofluorescence assay of MT2A in the dentate gyrus of the hippocampus of volcanogenic pollutants exposed mice **a** and reference area mice **b**. Scale bar: 100 µm. Magnification of image (**a**) and (**b**), respectively. **c**, **d**. Scale bar: 20 µm. Note the MT2A positive staining inside different dentate gyrus cells of exposed animals (**e**). Scale bar: 10 µm

**Fig. 3:**
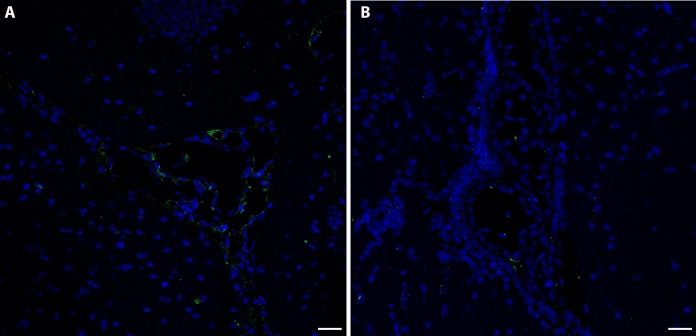
Presence of MT2A in the choroid plexus of mice from Furnas (**a**) and Rabo de Peixe (**b**). Note a difference in the expression of this metallothionein between both groups. Scale bar: 20 µm

Immunoreactivity to MT-2A in the brain of mice captured in the village of Furnas was greater than in the brain of those trapped in Rabo de Peixe. Particularly, immunoreactivity was located in the dentate gyrus of the hippocampus, both in its molecular and granular layers. In the exposed animals, a higher MT-2A labeling was observed in the border area between the molecular layer and the granular layer of the dentate gyrus, known as the subgranular zone, and in the white matter (Fig. 2a–c), than in the Rabo de Peixe animals (Fig. 2b–d). In addition, positive MT-2A labeling was also observed in the cytoplasm of some cells of the molecular layer (Fig. 2e) of the mice captured in Furnas. Animals chronically exposed to volcanic contaminants also showed significant MT-2A metalloprotein labeling in the choroid plexuses of the brain, in both the cytoplasm of epithelial cells and the blood vessels (Fig. 3a). However*, Mus musculus* individuals captured in Rabo de Peixe showed hardly any MT-2A labeling in the brain choroid plexuses (Fig. 3b).

**Fig. 4:**
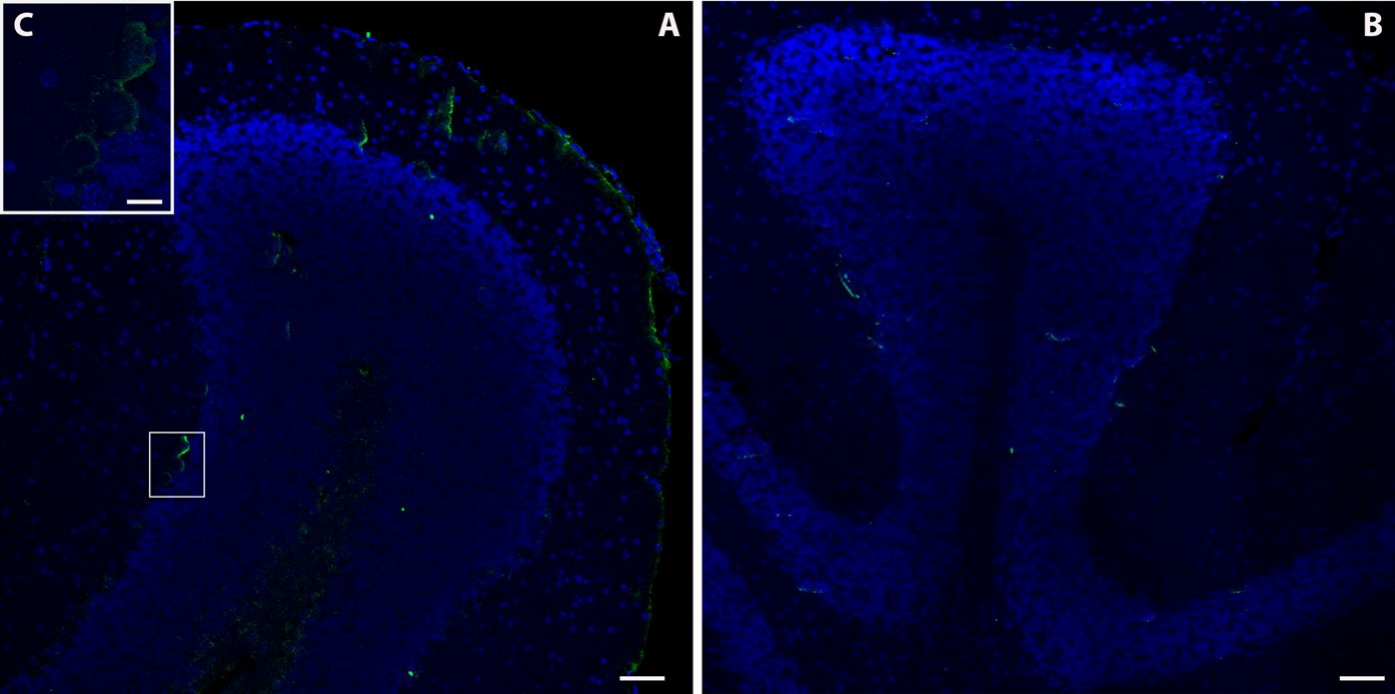
Photomicrograph of the immunoreactivity to MT2A in the cerebellum of Furnas **a** and Rabo de Peixe **b** individuals. Scale bar: 50 µm. Observe the presence of this metallothionein isoform inside Purkinje cells of those animals chronically exposed to volcanogenic pollution (**c**, inset). Sacle bar: 10 µm

Finally, the cerebellum of animals from the Furnas village showed a strong MT-2A immunoreactivity (Fig. 4a), particularly located in the white matter of the cerebellum or *arbor vitae*, which it was also observed in the meninges and inside some Purkinje cells (Fig. 4c). Weaker immunoreactivity was found in cerebellum of those from Rabo de Peixe (Fig. 4b).

## Discussion

Currently, heavy metal pollution is one of the greatest threats to the environment, capable of degrading the quality of water, soil, air and also affecting human health due to metal persistence, high toxicity and bioavailability (Yang et al., 2022). Although this type of pollution is closely related to anthropogenic sources, it can also occur due to natural causes, including volcanic activity. Volcanic contaminants, particularly from non-eruptive activity, yet present an underestimated risk to the human populations living in their vicinity. They emit various products that can be hazardous to human and animal health, triggering lesions especially in the nervous, respiratory and cardiovascular systems. For the first time, quantitative data is collected on the accumulation of mercury and other heavy metals in the brains of individuals chronically exposed to a non-eruptive volcanic environment.

Our findings suggest that the existence of volcanic activity on the island of Sao Miguel is responsible for the high amount of heavy metals such as Hg, Cd and Pb found in the brains of Furnas animals and the subtile presence of these elements in the brain of those captured in Rabo de Peixe, since neither location shows evidence of heavy industry or other forms of anthropogenic contamination. As for Hg, the chronically exposed mice had a sixfold higher concentration in the brain than the animals from the reference area. This quantitative result reinforces our previous reports in which the presence of inorganic mercury accumulations in different regions of the CNS was demonstrated by means of auto-metallography techniques (Navarro-Sempere et al., 2021b, 2022). Our results suggest, once again, the knowledge that the brain is one of the main target organs for mercury compounds, particularly regarding Hg^0^.

As discussed above, mercury, in its gaseous form, is one of the main metals released from volcanic systems. Hg^0^ enters by respiratory system, travels through the bloodstream and can cross the blood–brain barrier (BBB). Inside cells, this form of mercury is oxidized by the catalase-hydrogen peroxide pathway to Hg^2+^, which is considered the toxic form of this element (Aschner & Aschner, 1990; Clarkson & Magos, 2006; Fernandes Azevedo et al., 2012). Since Hg^2+^ cannot leave the cell, it is reduced back to Hg^0^ and travels cell to cell, in a process likely involving the superoxide anion and the coenzymes NADH and NADPH (Ogata et al., 1987).

Furthermore, in the current study we have also observed the presence of mercury in the brains of mice from the reference area, although in much smaller quantities probably due to the island’s own volcanic activity.

In addition to mercury, quiescent volcanoes release other heavy metals such as arsenic (As), Cadmium (Cd), Aluminum (Al), Rubidium (Rb), Lead (Pb), Magnesium (Mg), Copper (Cu) and Zinc (Zn). In the case of Furnas volcano Cd, Cu, Pb, Rb and Zn are normally present in volcanic emissions and their high bioavailability has been evidenced by their high concentrations in the scalp of the inhabitants of Furnas village (Amaral et al., 2008). Regarding these metals, in the present work, our results showed significant differences only for Pb and Cd, with *Mus musculus* individuals captured in Furnas having higher concentrations of these heavy metals in the brain compared to animals from the reference area. As pointed out by Amaral et al.,(2007), the presence of these heavy metals in mice may be due to the ingestion of food or soil rich in these metals. However, in that study, the animals also showed higher concentrations in the lungs, thus confirming the inhalation as a route of entry. This inhalation route has also been confirmed for Hg^0^ in animals inhabiting Sao Miguel Island, specifically in the Furnas region (Camarinho et al., 2021).

As for Pb, about 90% of lead particles present in the air are absorbed and retained by the body (Tokar et al., 2013). Pb particles that reach the lung can be phagocytosed by resident macrophages and the acidic environment created in the phagosomes is conducive to the release of Pb^++^ ions. These ions travel through the bloodstream and can cross the BBB by passive transport with the help of the calcium-dependent ATPase pump (Iqubal et al., 2020). In the brain, this metal accumulates mostly in the hippocampus and cortex (Bradbury & Deane, 1993). The overall effect of lead accumulation in the CNS is neuronal hyperexcitability and oxidative stress, which favor the occurrence of neuroinflammatory events such as activation of microglia and overexpression of proinflammatory cytokines such as nitric oxide synthase, IL-1 or TNF-α (Liu et al., 2012b). Previous work has shown that chronic exposure to volcanic environments triggers a neuroinflammatory response, activating microglia and increasing TNF-α in the dentate gyrus of the hippocampus (Navarro-Sempere et al., 2021a). These alterations caused to individuals living in the vicinity of a volcano are known to be involved in the pathophysiology of neurodegenerative pathologies such as Alzheimer's disease (Huat et al., 2019).

Cadmium, on the other hand, once inhaled enters the bloodstream through the lungs. Endothelial cells of the BBB present a series of transporters and receptors that facilitate the entry of this heavy metal from the bloodstream (Branca et al., 2020; Thévenod et al., 2019). Once inside the cells, Cd induces an oxidative stress situation, which sets in motion the cellular antioxidant machinery (Viaene et al., 2000). In the case of acute exposure to Cd, this machinery is sufficient to protect the CNS from Cd entry, but in circumstances of chronic exposure, the antioxidant defenses are weakened, increasing the permeability of the BBB and favoring the entry of more Cd into the brain (Shukla et al., 1996). It has been described that the first cells to be affected by the arrival of Cd into the CNS are astrocytes, since they are the intermediate between the BBB and the synapses. Astrocyte cells in response to chronic Cd exposure would increase the expression of glial fibrillary acidic protein (GFAP) (Khan et al., 2019) taking a more reactive role to protect the CNS. This fact is in agreement with results published in previous studies in which GFAP overexpression has been demonstrated in the dentate gyrus of the hippocampus of mice captured in Furnas village with respect to those inhabiting the reference area (Navarro et al., 2021).

Cells have developed some mechanisms to reduce the damage associated with heavy metal contamination, with toxic effects occurring when detoxification, metabolic and/or storage mechanisms are not able to counteract their uptake. Most heavy metals alter the delicate balance between ROS and the cells' antioxidant defense mechanisms, leading to an increase in ROS concentration. This creates an oxidative stress environment in cells that often leads to cell death (Balali-Mood et al., 2021). In this sense, metallothioneins are proteins that bind heavy metals, playing an important role in protecting against the toxic effects of these metals (Tokar et al., 2013). At the level of the nervous system, these proteins are present in glial cells and neurons and appear to have a protective effect against the neurotoxicity of some heavy metals such as lead, mercury and cadmium. It has been suggested that metallothioneins may be a way to prevent or treat neurodegenerative diseases (Juárez-Rebollar et al., 2017; Manso et al., 2011; Miyazaki & Asanuma, 2023; Samuel et al., 2021). In relation to this, MT2A is considered a key player in the maintenance of immune homeostasis (Jakovac et al., 2013) and its involvement as a neuroprotective factor has been described in different neurodegenerative diseases such as Parkinson's disease (Miyazaki et al., 2013), Alzheimer's disease (Chung et al., 2010), or amyotrophic lateral sclerosis (Brandebura et al., 2023). Our results show a strong expression of MT-2A in different regions of the CNS in those individuals who live chronically exposed to a volcanic environment, which would indicate that a detoxification process is taking place in the CNS cells due to the constant arrival and accumulation of heavy metals such as Pb, Cd or Hg. The difference in MTs expression between the two studied populations indicates, as has been described by Hidalgo et al., 2001, that the presence of Pb, Cd or Hg is capable of inducing the expression of metallothioneins in the mammalian brain. For this reason, MTs are considered biomarkers in the field of heavy metal-mediated environmental toxicology (Sakulsak, 2012). Moreover, not only the metals themselves induce the expression of these MTs, but also a neuroinflammatory environment marked by an increase in ROS and proinflammatory cytokines such as TNF-α or IL-6, could induce the overexpression of MTs (Kondoh et al., 2001; Penkowa et al., 2003). As noted above, the existence of this neuroinflammatory environment in the mice captured in the village of Furnas may be expressing MTs not only in response to heavy metals, but also to the neuroinflammation caused by other volcanic pollutants with which they coexist. The animals in the reference area (Rabo de Peixe) are not exposed to these other pollutants either, so, as Navarro-Sempere et al., 2021a reported, they did not show neuroinflammatory processes and, therefore, did not show an increase in MT expression.

Our findings reinforce the need for further studies on volcanogenic air pollution and its effects on the central nervous system, as it represents an important but unknown risk to the human worldwide population living in volcanic areas.

Authorship contribution.

Conceptualization was contributed by YS, MG, AN-S, PM-P, AR, PV, RC, GL,L-G; fieldwork and dissection were contributed by RC and AN-S; methodology was contributed by A N-S, PM-P, G-L and LG; data analysis was contributed by YS, MG, AN-S,GL and LG; writing—original draft preparation, was contributed by AN-S, YS, MG and GL; writing—review and editing, was contributed by AN-S, YS, MG, AR, PV,RC, PM-P,GL.
